# Reconciling Ecological and Engineering Design Principles for Building Microbiomes

**DOI:** 10.1128/mSystems.00106-19

**Published:** 2019-05-28

**Authors:** Hans C. Bernstein

**Affiliations:** a The Arctic Centre for Sustainable Energy, UiT—The Arctic University of Norway, Tromsø, Norway; b Faculty of Biosciences, Fisheries and Economics, UiT—The Arctic University of Norway, Tromsø, Norway

**Keywords:** community coalescence, ecological processes, environmental filtering, functional trait space, microbial community, microbial ecosystem, microbiome engineering, model system, priority effect, synthetic biology

## Abstract

Simplified microbial communities, or “benchtop microbiomes,” enable us to manage the profound complexity of microbial ecosystems. Widespread activities aiming to design and control communities result in novel resources for testing ecological theories and also for realizing new biotechnologies. There is much to be gained by reconciling engineering design principles with ecological processes that shape microbiomes in nature. In this short Perspective, I will address how natural processes such as environmental filtering, the establishment of priority effects, and community “blending” (coalescence) can be harnessed for engineering microbiomes from complex starting materials. I will also discuss how future microbiome architects may draw inspiration from modern practices in synthetic biology. This topic is based on an important overarching research goal, which is to understand how natural forces shape microbial communities and interspecies interactions such that new engineering design principles can be extracted to promote human health or energy and environmental sustainability.

## Abstract (precis)

Simplified microbial communities, or “benchtop microbiomes,” enable us to manage the profound complexity of microbial ecosystems. Widespread activities aiming to design and control communities result in novel resources for testing ecological theories and also for realizing new biotechnologies.

## PERSPECTIVE

Microbes rule our soils, our oceans, many of our bodily functions, our built environments, and our food, and many of us also find them fascinating. We have had a long and rich history of harnessing microbial ecosystems for engineering purposes, which is a practice being revolutionized by innovations borrowed from synthetic biology. Modern microbiome sciences have shown us that most microbes live in ultracomplex communities that are subject to spatial and temporal heterogeneity and often influenced by stochastic processes (1). In short, they are difficult to study if one aims to uncover the ecological processes that underpin community development and interspecies interaction. As we collectively begin to identify the connectivity between genome-encoded functions, community-level phenotypes, and environmental feedbacks for each ecosystem of interest, we are often faced with the challenge of lacking either the tools or resources typically available for model systems. Hence, while direct observation from native microbiomes is the most relevant approach for uncovering ecological processes, the results are often less conclusive than we may wish. Model microbial consortia, or “benchtop microbiomes,” have become popular tools for managing complexity and enabling access to state-of-the-art approaches such as genome engineering. Many of us have also developed a keen interest in learning to design, construct, and control microbial communities to perform complex functions that are not possible through traditional monoculture technologies (2, 3).

## MODEL SYSTEMS AND NEW SYNTHETIC BIOLOGY PLATFORMS

Why do we need model systems in biology, and what do they give us? Organismal biology has shown us through multiple famous examples that model systems such as the mouse or zebra fish accelerate research by providing a platform for standardized and reproducible investigations. Many major advances in microbiology are the result from a century or more of collective deep-dive research into model organisms: Escherichia coli, Bacillus subtilis, and Saccharomyces cerevisiae. But what about microbial communities? The criteria for building or adopting useful model microbial communities depend completely on the context of investigation. For example, many studies require explicit linkages to habitats such as soil, the human body, or marine euphotic zones. In these cases, it is difficult to imagine a single model microbiome ever becoming as widely adopted as the E. coli example has been for single-species investigations, because our collective efforts are spread across seemingly endless ecological categories. However, as we get better and faster at building ecosystem-relevant consortia, there may soon be an explosion in the number of model systems that can be shared and standardized to enhance collaboration. I support a vision—shared by others—in which cultivable microbial consortia will soon be archived and shared by a similar mechanism to modern day culture collections. Some challenges must be overcome for this to be realized; such as the ability to arrest or manage community-level adaptation. These efforts should, in turn, lead to new platforms, or “chassis,” for innovation and expansion in synthetic biology.

Other reasons that fundamental scientists have for building microbial communities are more system agnostic and typically seek to understand generalizable principles such as the emergence of higher-order properties or energetic drivers of metabolite exchange (2, 4). Architects of these communities often prioritize control of taxonomic and/or functional diversity over representation of natural ecosystems. Enhancing open access and collaboration should enable us to develop and implement standardized design strategies and modular parts that can be recycled to make microbiome engineering easier through each successive round. These efforts can learn from the advances made to organize and categorize genetic parts used by synthetic biologists (5). I hope for near-term developments in this area where microbial ecologists borrow from frameworks such as BioBrick (6), Addgene (www.addgene.org), and the Registry of Standard Biological Parts (http://parts.igem.org/).

## HARNESSING NATURAL ECOLOGICAL PROCESSES AS NEW ENGINEERING DESIGN PRINCIPLES

There are two common approaches—within the current state of the science—for developing model microbial communities: top-down selection of natural microbiomes toward representative consortia with reduced complexity (7) and bottom-up construction of consortia, which are predesigned assemblages based on hypotheses or specific engineering design principles (8–10). These approaches have been discussed and compared in a number of review and perspective articles (2–4, 11) and hence will not be covered in more detail here.

**FIG 1:**
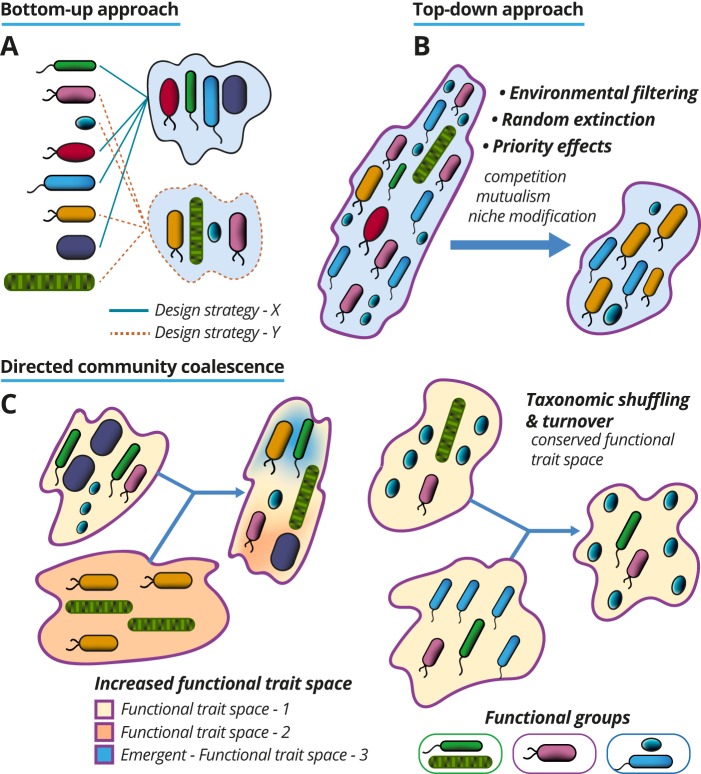
Microbiome engineering is an emerging field that draws from both synthetic biology and microbial ecology. Single species and controllable elements of the environment (such as those that can be maintained in cultivation chambers) can be used to build microbiomes via bottom-up approaches (A). In the bottom-up cases, it is easy to conceptualize the modular parts that can be used or reused, manipulated, and shared. However, whole communities and elements of their environment can also be harnessed as modular parts and used or reused as starting materials to select and/or dilute complex microbiomes down to more tractable communities. This top-down approach (B) can be tailored around specific ecological processes harnessed as engineering design principles. One potential frontier in microbiome engineering expands upon the idea of using whole communities (and environmental factors) as modular parts through controlled blending processes that can be defined as directed community coalescence (C). These approaches can be designed to capitalize on common processes that influence natural microbiomes to control/design relationships between functional trait space and taxonomic structure.

Microbiome engineering can also be practiced through hybrid approaches, where different complex communities and elements of their associated environments are mixed by means that enable the engineer to capitalize on natural ecological processes as design principles (Fig. 1). This is a promising area that should attract more interest as we become better at understanding how different ecological forces shape microbiomes. In nature, microbial communities and elements of their associated environments are routinely moved and blended in a distinct assembly process that has been termed “community coalescence” (12). An important concept that we apply to understand the outcome of community coalescence is that microbes interact and stabilize/destabilize newly blended communities by interacting as internally integrated units, as opposed to stochastic rearrangements of mixed species. I argue that some of the processes that are known to influence outcomes of community coalescence can be harnessed for constructing and controlling the structure and function of microbiomes. This can be conceptualized as an extension of the traditional bottom-up design approach—i.e., combination or recombination of parts—where different cultivable communities and aspects of their environments can become modular building materials. The power of this idea comes in part from the inclusion of environmental mixing (or lack of), which is fundamentally built into the concept of community coalescence.

Environmental mixing itself can be a controllable property and therefore can also be used as an engineering design tool. For example, careful control over environmental mixing can be harnessed to both design and control the degree of “environmental filtering,” which is the process by which an environment selects against certain species. This can be realized by preconditioning one community as a starting material prior to blending it with donor communities that have been conditioned to different environments and/or different selection forces. A “priority effect” is the ability for a given species to influence later stages of community structure and/or function. The assignment and utilization of priority effects may also be realized as an engineering design strategy that can again be controlled by preconditioning one community—or even monocultures—prior to the addition of another. One potential application would be successive challenges of a model community with new communities in an effort to strengthen priority effects that enhance resistance or resilience against invasion. An obvious application for employing this type of microbiome engineering applies to the growing interest in developing designer human microbiomes to combat or resist invasion by pathogens.

The “functional trait space” of a community can be defined as the number of measurable characteristics that affect the performance or fitness of individual members or the whole community (13). The ability to shape and maintain a desired functional trait space is—or will be—a primary goal for microbiome engineers. In some applications, it may be desirable to expand a microbiome’s functional trait space. This may be realized via directed community coalescence, specifically by blending communities that occupy very different trait spaces under similar environments and/or selection forces. In this case, one could expect the additive result to be a new microbiome that harbors more functional traits than any of the individual source communities (Fig. 1C). This prospective engineering process may become very interesting if and when we learn to design and build higher-order properties, where the number of functional traits exceeds the sum of those that are measurable from all source communities (2–4). In a similar way, a directed community coalescence approach may be developed to change taxonomic structures of microbiomes while maintaining functional trait space. This may be realizable by blending communities that have similar functional traits held by different taxa maintained under similar environments and selection pressures. The expectation would be that competition results in the replacement of taxa within available niches, a process called “turnover” (Fig. 1C). One exciting element of this approach will be to learn to control the relative contribution of deterministic processes to turnover (1): specifically, the ability to know when the blending of communities will reproducibly converge to the same taxonomic structures or diverge unpredictably based on random demographic events. When it is discovered that directed community assembly is driven by stochastic forces, then microbiome engineers may be able to capitalize on the process in similar ways that metabolic engineers have successfully used random mutation approaches (14). The adoption of random taxonomic shuffling techniques—within bounded functional trait spaces—may have significant advantages over rational design because of the inherent complexities of microbiomes and our current inability to disentangle relationships between taxa, genome-encoded functions, phenotypes, and environmental factors.

## OBSERVING NATURE OR ENGINEERING IT?

As a researcher that operates at the juxtaposition of microbial ecology and bioengineering, I often struggle with defining boundaries between the fundamental and applied aspects of my research on microbial communities. There is a common, yet seemingly artificial, compartmentalization of scientific culture, language, and approach that partitions the two practices of observing nature and engineering or reengineering it. Language and accepted definitions are the obvious and most difficult places to begin reconciling our understanding of engineering design principles with ecological processes. There are so many examples, so I will give just one here. What is the definition of a community? Traditionally, “communities” have been defined as multispecies assemblages where members live together in a contiguous environment and interact. However, synthetic ecology has already challenged this definition by assembling microbial consortia from metabolically engineered and functionally differentiated members of the same species (15). Are these still communities or are they monocultures? This relates to a fundamental question for ecologists, which is whether community member differentiation should be defined by taxonomy or function. Another challenge for reconciliation comes into play when trying to define a “system.” Chemical and biological engineers can easily define a system by drawing physical boundaries to distinguish inputs and outputs of conserved quantities: mass, energy, and momentum. While these principles certainly apply to any ecosystem, ecologists—especially molecular ecologists—often encounter more difficulty in defining their systems. How does one draw a boundary around a microbial community? By respecting the frameworks given to us by community ecologists, we must account for several factors when attempting to define a microbial community (11). There are taxonomic and genomic bases for defining the boundaries of microbial communities, which rely solely on sequenced information and are not defined by physical spaces or mass/energy balances. There is also the concept of interaction. Is it appropriate to group microbes into the same community if they inhabit the same space but never interact or influence each other? In this spirit, I will channel a perspective put forth by Konopka (11) by saying that it is critically important for both ecologists and engineers to explicitly articulate their definitions with respect to each individual research effort or engineering design goal. This should not be at odds with generalizability, and definitions must still be chosen with care to avoid isolating scientific advancements. Clear, context-specific communication coupled with tolerance for multidiscipline ambiguity will lead to more rapid exchange of ideas between ecologists and engineers. This in turn, will open new doors for us to begin using natural, living materials—including entire microbial ecosystems—as the unit operations for entirely new fields of engineering. I also believe that these directions shall ultimately play roles in the most imaginative future applications, including combating some effects of climate change, stabilizing marine ecosystems, and enabling humans to colonize extraterrestrial habitats.
